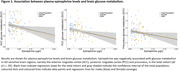# Circulating stress hormones and multimodal measures of brain and cognition in older adults: Cross‐sectional findings from the AGE‐WELL cohort

**DOI:** 10.1002/alz.088827

**Published:** 2025-01-09

**Authors:** Maxie Liebscher, Silke White, Edelweiss Touron, Florence Mézenge, Anne Chocat, Laurent Coulbaut, Denis Vivien, Vincent De la Sayette, Natalie L Marchant, Gael Chételat, Olga M. Klimecki, Géraldine Poisnel, Miranka Wirth, Medit‐Ageing Research Group

**Affiliations:** ^1^ German Center for Neurodegenerative Diseases (DZNE), Dresden Germany; ^2^ Normandie Univ, UNICAEN, INSERM, U1237, PhIND "Physiopathology and Imaging of Neurological Disorders", NeuroPresage Team, GIP Cyceron, Caen France; ^3^ Département de Recherche Clinique, CHU Caen‐Normandie, Caen France; ^4^ Service de Neurologie, CHU Caen‐Normandie, Caen France; ^5^ Division of Psychiatry, University College London, London UK; ^6^ Friedrich Schiller University Jena, Jena Germany

## Abstract

**Background:**

Increased stress, a proposed risk factor for Alzheimer’s disease (AD), is associated with increased brain and cognitive vulnerabilities in older populations, which may be different in women and men.

**Objective:**

To examine cross‐sectional associations between circulating stress hormones (epinephrine, norepinephrine, cortisol, dehydroepiandrosterone sulfate (DHEAS), and DHEAS/cortisol ratio) and multimodal measures of brain health and cognition sensitive to AD.

**Method:**

132 cognitively unimpaired older participants without clinical depression (age = 74.0 ± 4 years, females: n = 80) were included from the Age‐Well baseline dataset. Stress hormones were measured in overnight fasting blood serum (cortisol, DHEAS) and plasma (epinephrine, norepinephrine). The association of stress hormone levels with glucose metabolism and perfusion in AD sensitive brain regions, including the anterior and posterior cingulate cortex (ACC, PCC), insula and, precuneus, along with neocortical amyloid deposition and cognitive markers, including memory and Preclinical Alzheimer's Cognitive Composite‐5 (PACC5), was assessed. Linear regression models with and without stratification by sex adjusting for covariates of age, sex, education, subclinical anxiety, and depression were conducted.

**Result:**

In the total cohort, higher epinephrine was associated with lower glucose metabolism (Figure 1) in the ACC (adj.‐β = ‐0.26, p = .027), PCC (adj.‐β = ‐0.32, p = .006), and precuneus (adj.‐β = ‐0.27, p = .021) and lower perfusion in the PCC (adj.‐β = ‐0.23, p = .013). Sex‐stratified analyses showed interactions (all p’s < .1): In males (but not in females), higher cortisol was associated with lower episodic memory (adj.‐β = ‐0.33, p = .02), short‐term memory (adj.‐β = ‐0.32, p = .014) and PACC5 scores (adj.‐β = ‐0.28, p =.04), suggesting a stress‐related vulnerability in the cognitive system of men. Stress biomarkers were not associated with neocortical amyloid deposition (all p’s ≥ .1).

**Conclusion:**

Our results demonstrate the involvement of stress hormones, particularly epinephrine and cortisol, in increased vulnerability of the brain and cognition in older adults and the manifestation of sex specificities in this context. The role of stress on brain and cognitive health and related sex differences needs to be considered in intervention programs.